# Expert Perspectives on Managing Iron Deficiency in People with CKD and/or HF

**DOI:** 10.3390/jcm15041676

**Published:** 2026-02-23

**Authors:** Sunil Bhandari, John G. F. Cleland, Fozia Z. Ahmed, Fraser J. Graham, Matt Hall, Paul R. Kalra, Philip A. Kalra, Kate I. Stevens, David C. Wheeler, Simon G. Williams, Dora. I. A. Pereira, Marco Soscia, Harry Lewis, Imogen Taylor

**Affiliations:** 1 Department of Renal Medicine, University Teaching Hospitals NHS Trust, Hull HU3 2JZ, UK; sunil.bhandari@nhs.net; 2 School of Cardiovascular & Metabolic Health, University of Glasgow, Glasgow G12 8QQ, UK; john.cleland@glasgow.ac.uk; 3 Department of Cardiology, Manchester University NHS Foundation Trust, Manchester M13 9WL, UK; 4 School of Health and Wellbeing, University of Glasgow, Glasgow G12 811, UK; fraser.graham@glasgow.ac.uk; 5 Renal and Transplant Unit, Nottingham University Hospitals NHS Foundation Trust, Nottingham NG5 1PB, UK; matthew.hall10@nhs.net; 6 Department of Cardiology, Portsmouth Hospitals University NHS Trust, Portsmouth PO6 3LY, UK; paulkalra@doctors.org.uk; 7 Faculty of Biology, Medicine and Health, University of Manchester, Manchester M13 9QL, UK; philip.kalra@nca.nhs.uk; 8 Renal Department, Queen Elizabeth University Hospital, Glasgow G51 4TF, UK; kate.stevens@nhs.scot; 9 Centre for Kidney and Bladder Health, Royal Free Campus, University College London, London NW3 2PF, UK; d.wheeler@ucl.ac.uk; 10 Division of Cardiovascular Sciences, University of Manchester, Manchester M13 9QL, UK; simon.williams2@mft.nhs.uk; 11 CSL Vifor, Maidenhead SL6 8AA, UK; dorapereira200@gmail.com (D.I.A.P.); marco.soscia@viforpharma.com (M.S.); 12 Initiate Consultancy, Alderton NN12 7LS, UK; j.mumford@initiateconsultancy.com

**Keywords:** iron deficiency, heart failure, chronic kidney disease

## Abstract

**Background:** Iron deficiency (ID) is common among people with chronic kidney disease (CKD) and/or heart failure (HF). Despite the additional burden ID causes among people with CKD and HF, there is considerable uncertainty surrounding the best way to diagnose it and, subsequently, identify who is most likely to benefit from receiving iron therapy. **Methods:** This manuscript reports the markers and thresholds used in ID diagnosis, treatment, and management in the UK by nephrologists and cardiologists who manage people with chronic kidney disease or heart failure, as well as investigating future challenges and questions that remain unanswered. The research involved three stages: an online questionnaire, individual interviews, and a panel meeting, which discussed the findings from the first two stages. **Results:** The panel concluded that there is no robust definition of iron deficiency that can be applied to chronic kidney disease and heart failure. Existing methods of diagnosing iron deficiency come with various problems; a transferrin saturation of <20% is the most popular, but it is not regarded as a perfect solution. Transferrin saturation is also the most popular way of assessing the success of iron deficiency treatment. Clinicians generally do not vary treatment regimens based on severity or subgroups. There are large variations in monitoring and the ability to administer iron therapy in secondary care. **Conclusions:** There is a clear need to consolidate current approaches to diagnosing and treating iron deficiency in people with chronic kidney disease and/or heart failure. Simple markers and thresholds, and simple strategies to implement them are required.

## 1. Introduction

Iron deficiency (ID) is estimated to affect up to 1 billion people worldwide [1] and is common among people with chronic kidney disease (CKD) and/or heart failure (HF). ID is uncommon in patients who have a truly normal haemoglobin (>1 g/dL above the WHO threshold). Many patients with borderline anaemia have ID. When ID is severe, iron deficiency anaemia (IDA) is common. Anaemia may be due to causes other than ID. Accordingly, haemoglobin and measures of ID should be interpreted in tandem.

Iron is an essential component of haemoglobin and myoglobin, which are essential for oxygen transport and uptake by cardiac and skeletal muscle. Iron is also a key component of the mitochondrial electron transport chain and, therefore, oxygen utilisation and energy production. Thus, ID is especially deleterious for organs with high energy demands, including the brain and heart [2,3,4], affecting general wellbeing, physical performance, and cognitive function [5].

The body contains only a small amount of iron, about 5–6 g. Too much iron is toxic and there is no system to excrete it from the body, so systems have evolved to limit iron absorption. For normal daily physiological activities, the primary source of iron is macrophages that have processed senescent red blood cells and removed the iron. Ferritin acts as a storage site from which iron can flow in and out. Hepcidin, a hormone produced by the liver, is the master regulator of iron homeostasis. It is secreted when iron stores are replete and binds to the iron transporter ferroportin to reduce further absorption of iron from the duodenum. In conditions such as CKD or HF, and in chronic inflammation more generally, hepcidin is elevated due to inflammatory cytokines such as interleukin-6 (IL-6). This leads to the body becoming unable to absorb iron from the duodenum or release it from the retico-endothelium, hence leading to iron deficiency [6,7,8].

In addition, iron deficiency anaemia in CKD may result from blood loss from repeated blood sampling, shortened red blood cell survival from various uraemic toxins, or the impact of hyperparathyroidism on bone marrow [9,10,11,12]. The mechanism of iron deficiency is similar in both HF and CKD, and many HF patients have a low estimated glomerular filtration rate, which may in part be due to CKD (although this label may be unhelpful when managing patients) [13,14]. As such, there is significant overlap in the way they are treated in clinical practice.

ID can be classified as either absolute or functional. In absolute ID, total body stores of iron are low due to dietary deficiency, reduced absorption, and/or increased losses due to bleeding. ID may also be caused by heightened iron demand during pregnancy or treatment with recombinant human erythropoietin [15,16]. People with absolute ID usually have low hepcidin levels, but with CKD and HF, increased inflammation may prevent suppression of hepcidin secretion. In functional ID, the total amount of iron in the body may be normal or even increased, but iron is sequestered in the reticuloendothelial system with reduced access for physiological use [17]. Hepcidin will often be at normal or increased levels in these cases, despite the low availability of iron. Many people have features of both absolute and functional ID.

People with CKD and/or HF often have coronary artery disease and will be prescribed aspirin, which increases the risk of occult and overt gastrointestinal (GI) bleeding. People with atrial fibrillation will usually be prescribed oral anticoagulants that may increase the risk of major bleeding. Many people on aspirin are prescribed treatments to relieve dyspepsia and to reduce the risk of GI bleeding; however, reducing gastric acidity impairs iron absorption. People with CKD and/or HF are often elderly and at increased risk of GI cancers.

In both CKD and HF, and indeed more widely, ID has been linked to increased morbidity and mortality [18,19,20,21,22,23]. It is unclear to what extent ID contributes to worse outcomes or is simply a marker of more severe underlying disease. People with CKD who are anaemic are more likely to have coronary artery disease, congestive heart failure, and diabetes [6,24]. In HF, ID is associated with reduced exercise capacity, impaired quality of life, and a higher risk of hospitalisation [25,26].

Despite the additional burden ID causes among people with CKD and HF, there is considerable uncertainty surrounding the best way to diagnose it and, subsequently, identify who is most likely to benefit from receiving iron therapy. Often, diagnostic assessments are not performed at all; patients who do receive a diagnosis may not receive treatment. As such, management of ID is often sub-optimal [27].

This research aimed to report the markers and thresholds used in ID diagnosis, treatment, and management in the UK by nephrologists and cardiologists who manage people with CKD or HF, as well as to investigate future challenges and questions that remain unanswered. It highlights the need to consolidate current approaches to diagnosing and treating ID in people with CKD and/or HF, and to develop simple markers and thresholds.

## 2. Materials and Methods

This research involved three stages: a questionnaire (Stage 1), individual interviews (Stage 2), and a panel meeting involving all participants (Stage 3).

The questionnaire was created using Qualtrics, an online survey platform, ensuring the anonymity of individual participants. The online questionnaire had a series of closed questions or statements to express their level of agreement or disagreement on a 5-point scale (“completely agree,” “agree,” “neutral,” “disagree,” and “completely disagree”). A copy of the questionnaire can be found in the Supplementary Materials.

Results from the questionnaire, which required further discussion, were raised in subsequent individual interviews. In Stages 1 and 2, many of the questions and statements were influenced by unanswered questions from the ‘KDIGO Controversies Conference on Optimal Anemia Management in CKD’ [28].

The final stage, the panel meeting, included presenting back the findings from the first two stages, analysing the results and delving into further details for each topic. During the meeting, the online Slido platform was used to run polls, providing a quick overview of the full panel’s views. Participants who disagreed or had opposing views to the rest of the panel were encouraged to provide reasons for their choice, followed by discussions with the whole panel. The research process was an iterative one, with each stage building upon the last by focusing on areas of uncertainty or disagreement between the panellists. In the consensus meeting, consolidated findings from the previous two stages were presented, with the Slido polls used to gauge the level of overall agreement with these findings among the panel. As such, most of this article focuses on the findings of the final stage.

### Participants

5 cardiologists and 5 nephrologists took part in the panel, all of whom had significant experience managing iron deficiency: a combined total of 194 years (range: 7–30 years; median: 18.5 years), which was generally well-balanced between the cardiologists (total: 80; range: 7–28; median: 15) and nephrologists (total: 114; range: 17–30; median: 25). It was estimated that, as a group, they currently manage a total of 1995 (range: 30–500) people with ID and see a total of 872 (range: 15–500) new patients with ID annually. The selection of the panellists was primarily determined by their experience in managing ID and standing within the fields of cardiology and nephrology, as it was felt that this would allow for a broad overall perspective while retaining the ability of a smaller group to produce detailed qualitative insights.

## 3. Results

### 3.1. Burden of Iron Deficiency

The impact of ID on quality of life is difficult to assess because symptoms are non-specific and overlap with those of other diseases, particularly CKD and HF. Nevertheless, the panel unanimously agreed that ID has a negative impact on quality of life, particularly if it is also associated with anaemia. Whether iron deficiency without anaemia has a major impact on quality of life is less certain, but more research is needed [4,29]. Some clinicians mentioned that the effect of ID on a person’s daily life might depend on their functional demands, i.e., those who are more physically active might be more impacted.

The people managed by the panel often had comorbid conditions in addition to CKD and HF. Treatment for these conditions may cause side effects (e.g., beta-blockers may increase fatigue), making the process of attributing individual symptoms to ID even more difficult.

The following symptoms were mentioned as being particularly common, albeit not specific to ID:

- Fatigue and lethargy;
- Breathlessness;
- Worsened sleep quality;
- Decreased mental wellbeing;
- Impaired cognitive function.

### 3.2. Diagnosing Iron Deficiency

There is no standardised laboratory definition of iron deficiency. Similarly, although clinicians in the panel cited various guidelines that covered the diagnosis of ID, there is no consensus on which to use for this purpose [30,31,32,33,34,35,36]. The panel reported numerous potential methods for diagnosing ID but concluded that there was no robust definition that could be applied to people with CKD or HF.

**Figure 1:**
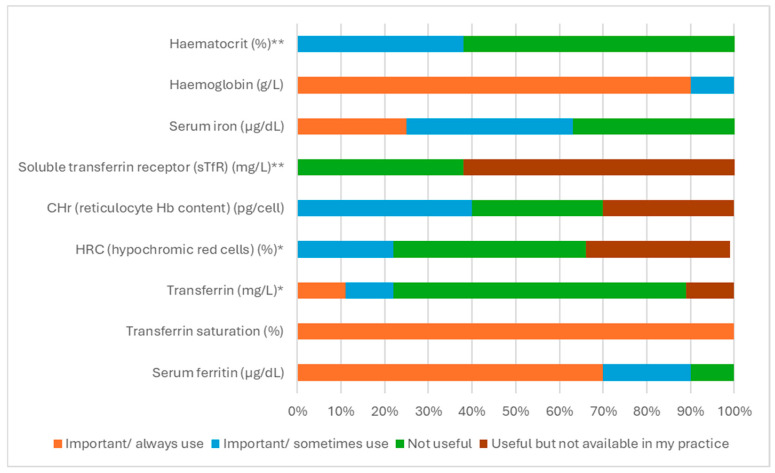
Parameters used to evaluate iron deficiency (*n* = 10, unless otherwise stated; * *n* = 9, ** *n* = 8).

All completed questionnaires mentioned transferrin saturation (TSAT) < 20% as an ‘important’ marker of ID, with many emphasising its simplicity and binary nature as a key factor in its usage. TSAT < 20% is mentioned in guidelines published by the National Institute of Health and Care Excellence (NICE), the European Society of Cardiology (ESC), and the UK Kidney Association, among others. When asked in the questionnaire to rank the main diagnostic markers in order of preference for clinical use, TSAT was ranked most highly by far; see Figure 1. Serum ferritin concentration was ranked much lower. Despite its widespread acceptance, however, many clinicians were keen to emphasise that they would not look only at TSAT levels, showing that, despite its popularity, TSAT is not regarded as the sole solution to the problem of diagnosing ID.

Discussions around ferritin featured heavily during both the individual interviews and the panel discussion, with several panellists saying they used ferritin in conjunction with TSAT when diagnosing someone. One clinician who declared TSAT < 20% to be “the clear, binary, and widely accepted definition” nevertheless also made clear they believed ferritin could play a part in determining whether someone had ID. Nephrologists tended to be more positive towards the use of ferritin.

Overall, however, the panel was doubtful regarding ferritin’s utility for the diagnosis of ID unless values were very low. In the 1970s, a ferritin level of <10 µg/L was said to indicate iron deficiency; the World Health Organisation (WHO) has suggested a threshold of 15 µg/L for adults, but a threshold of 30 µg/L is widely used [37]. High hepcidin levels affecting iron repletion mean that in inflammatory conditions, the WHO threshold is higher, up to 70 µg/L [38,39]. The lack of clarity around thresholds fed into wider questions surrounding how reliably ferritin can identify ID, with several clinicians noting how the clinical community is moving away from ferritin and embracing other methods.

Haemoglobin was also regularly cited by the panel. On the whole, it was viewed much more positively than ferritin. There was some disagreement over the diagnostic threshold for anaemia, with answers ranging from 100 g/L, the old WHO threshold for haemoglobin in a healthy individual, to the current WHO threshold (<120 g/L for women and <130 g/L for men), to advocates for ‘borderline’ anaemia (0–10 g/L above WHO thresholds), to 150 g/L, which has been used as a ‘normal’ threshold in CKD [40,41,42]. Nevertheless, 90% (9 of 10) of clinicians said they always considered haemoglobin when diagnosing ID; during the group meeting, it was agreed that it was a “key measure” alongside TSAT. One clinician noted that they would use it first as a measure for IDA, before proceeding to check ferritin and TSAT if levels were normal.

Some panellists were in favour of serum iron as a better measure than TSAT. Others considered that serum iron was too subject to diurnal variation, but advocates of serum iron suggested that similar diurnal variation occurred with TSAT and to a lesser extent haemoglobin and ferritin. Transferrin has been shown to have a strong inverse relationship with ferritin, meaning that when serum ferritin is high, serum transferrin will be low and easy to saturate even when serum iron concentrations are low, potentially leading to a missed diagnosis of ID [43]. Similarly, whereas ferritin increases in cases of inflammation, transferrin concentrations tend to decrease, and in CKD patients, they can also decrease due to renal leak and loss to dialysis [44]. A short summary from one panel member was “stop all the confusion with ferritin—TSAT and haemoglobin, that is what we need to know”.

Several additional tests were mentioned by clinicians when prompted, although there was broad scepticism about their usefulness. Haematocrit, hypochromic blood cells, and reticulocyte haemoglobin (particularly among nephrologists), soluble transferrin receptor, and hepcidin were all discussed, although the evidence for their use in clinical practice and ability to predict the effects of iron therapy was found to be lacking. Ultimately, 90% of the panel agreed that most additional tests are not useful for providing additional insights beyond TSAT, ferritin, and haemoglobin when diagnosing ID. Some strongly advocated simple screening for blood in faeces and urine to identify sources of chronic blood loss that might reflect underlying cancers, although the utility of these practices is in studying the aetiology of ID, not diagnosing it.

**Figure 2:**
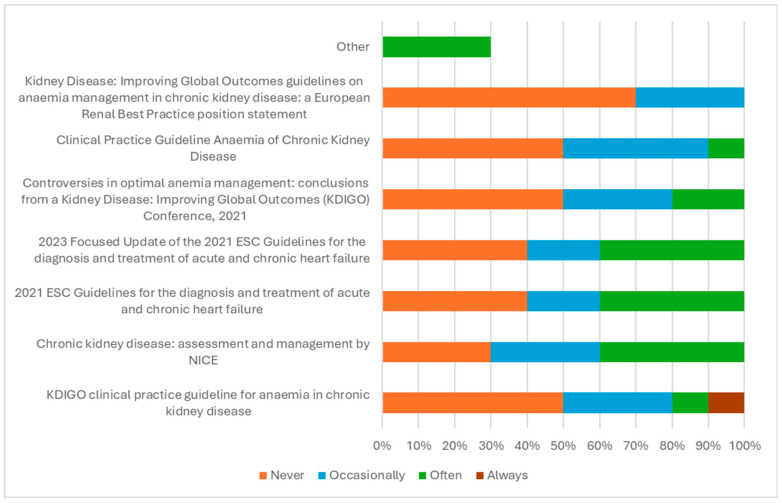
Guidelines used in the diagnosis of iron deficiency (*n* = 10) [30,31,32,33,34,35,36].

Figure 2 displays the wide variation in diagnostic guideline use among the panel: none were used by more than 70% of the panellists, and only one clinician cited guidelines they ‘always’ used.

Clinicians found diagnosing absolute and functional ID to be difficult. The symptomologies of absolute and functional ID significantly overlap, and despite the existence of definitions based on blood tests, the clinical differences between them are small. As such, they may represent two ends of a continuous spectrum rather than being distinct entities. Clinicians were also largely in agreement that the absolute/functional distinction does not influence treatment, although evidence has shown that SGLT2 inhibitors might theoretically have a greater effect on functional ID due to their ability to reduce inflammatory cytokines [45]. Conversely, oral iron supplementation may be less effective in functional ID because high hepcidin levels can interfere with iron absorption [46]. Nevertheless, most panellists (67%; 6 of 9) agreed that there was no current need to distinguish absolute and functional iron deficiency in a clinical setting.

Nephrologists emphasised the split between people receiving dialysis and those not requiring dialysis: dialysis is associated with substantial, regular blood loss, leading to absolute ID in most cases.

### 3.3. Treatment of Iron Deficiency: Iron Deficiency Versus Anaemia

Most (80%; 8 of 10) panellists agreed that people should be screened for iron deficiency, whether or not they had anaemia. Most panellists (90%; 9 of 10) also agreed that ID should be treated whether or not the person had anaemia; this was despite their belief that there was a paucity of trial evidence that people without anaemia benefit from iron supplements. Few people with severe CKD are not anaemic. However, one heart failure specialist estimated that 30% of people with heart failure and a TSAT < 20% were not anaemic, and called for further investigation into whether this group benefits from iron therapy. Another clinician stressed that the full impact of iron deficiency on the body, whether on enzymes, mitochondria, or elsewhere, is still unknown. However, the presence of anaemia is a marker that many other physiological systems may be iron deplete.

Most of the panel considered that the most solid evidence for the benefit of iron therapy was in people with IDA. However, one panellist suggested that the definition of anaemia was too restrictive and should be about 1 g/dL higher than the WHO definition [42]. There is a clear need for further research on ID and anaemia.

### 3.4. Treatment of Iron Deficiency: Patient Management

According to the panel, the vast majority of treatment for ID takes place in secondary care. There was a large variation in how often retesting takes place. People on haemodialysis might have their iron indices checked every 1 to 3 months, whereas heart failure specialists might reassess iron status every 6 to 12 months. Many specialists discharge stable patients to general practitioners, where their iron status may not be routinely checked.

When asked about potential equity issues, several clinicians noted that many people with ID are elderly, something which is true in both CKD and HF: the elderly’s health concerns may be falsely attributed to their age rather than their specific medical condition. A similar concern was raised about socioeconomically disadvantaged groups; they, as well as ethnic minority groups, were cited as being at risk of disengagement from healthcare settings. Many clinicians additionally pointed out that the availability of ID care varies from hospital to hospital, with some centres having limitations on their ability to prescribe certain treatments. These issues (as well as the fact that the symptoms of ID are non-distinct) were thought to contribute to the general under-diagnosis and under-treatment of ID.

As well as varying in their ability to administer iron therapy, many hospitals vary in their policies towards managing ID. Clinicians unanimously stressed the importance of having well-organised services with shared resources across departments. For people on haemodialysis, it is relatively easy to include regular monitoring of iron status and iron therapy administration into haemodialysis visits. People with CKD or HF under regular specialist review are likely to have haemoglobin and iron status checked regularly. However, when they are discharged back to primary care, specialists may not give clear recommendations about the importance of checking haemoglobin and iron status periodically, and even if they do, there is great variability in whether recommendations are followed.

Care plans that include recommendations for monitoring intervals for laboratory tests should be developed, implemented, and audited across primary and secondary care. Several panellists stressed the importance of having a single protocol for delivering iron therapy across hospital departments, something which is not always achieved.

**Figure 3:**
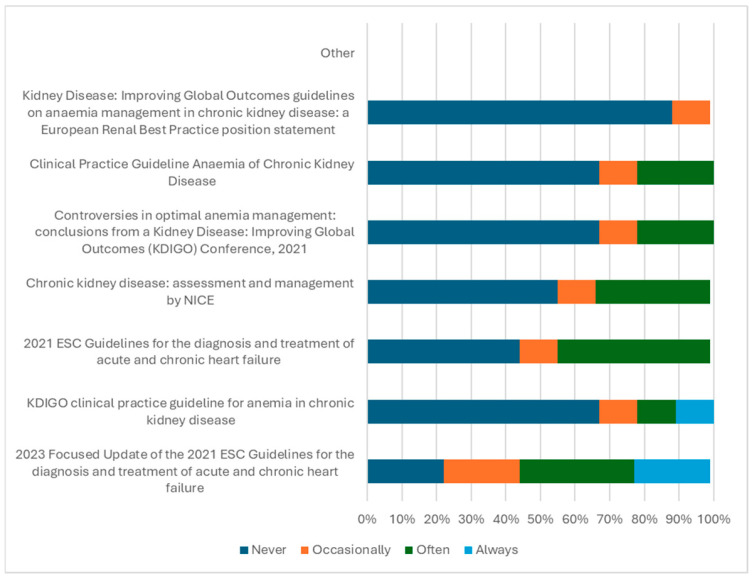
Guidelines used in the treatment of iron deficiency (*n* = 9) [30,31,32,33,34,35,36].

ESC guidelines were the most popular for guiding treatment among the panel, as shown in Figure 3. Most panel members stated they would initiate iron therapy treatment immediately upon diagnosing ID.

### 3.5. Treatment of Iron Deficiency: Treatment Thresholds

TSAT was the most popular way to assess the success of treatment, with an optimal range of 20–40% being cited several times. Haemoglobin was also sometimes used. Again, the panel took a more negative view of the use of ferritin in this context; concerns were raised around large variations in measurements depending on the time at which the measurement was taken. Some clinicians, especially cardiologists, said they did not use treatment targets other than ensuring TSAT was >20%, several of whom pointed to a lack of evidence that higher TSAT is associated with improved outcomes.

In general, clinicians in both CKD and HF did not vary their treatment regimens based on how severe someone’s ID was, with 80% of the panel (8 of 10) saying they did not define ID by severity. Indeed, most framed their decisions regarding treatment as binary—to treat or not to treat—with only the people who met the threshold for ID diagnosis receiving iron supplementation. Clinicians that did vary treatment usually did so based on the degree of a person’s anaemia. Someone with anaemia and a borderline TSAT might receive intravenous iron therapy, but those who did not have anaemia might receive oral iron or no iron repletion therapy at all. Some clinicians said they might treat someone with no or only mild symptoms slightly differently, either by using oral iron as a first option (as opposed to IV) or by retesting to confirm ID before initiating treatment. Most clinicians, however, stuck fairly rigidly to the attitude of not defining severity at all; ID was either present and should be treated or absent, with any decision on treatment waiting until the next test.

Treatment did not vary much among other subgroups either, although there were some exceptions. Some nephrologists thought that people with CKD were at higher risk of anaemia if they had diabetes and therefore required closer monitoring. Clinicians were also concerned about older and more frail people, as well as those with deep vein thrombosis, stroke, or malignancies. The panel stressed that they would always tailor treatment to the individual. For dialysis patients, the decision to re-dose with iron is typically based on a combination of tests as well as a more holistic view of the patients’ needs. Overall, clinicians still considered a personalised approach to treatment to be important despite the overall lack of treatment variation.

Nephrologists were unanimous in saying that there is a need to differentiate targets based on whether a person is on erythropoietin-stimulating agents (ESAs). The haemoglobin target for people with kidney disease on ESA therapy is 100–120 g/L [31,35,47]. ESA therapy can also affect non-Hb targets such as TSAT and ferritin, and targets for these may differ if the aim is ESA sparing, particularly in people undergoing dialysis. No specific values were mentioned for those markers.

## 4. Discussion

Cardiologists and nephrologists face a myriad of challenges when diagnosing and treating iron deficiency. There is substantial disagreement on the best approach to selecting people with CKD or HF who are likely to benefit from iron therapy, and little discussion on whether thresholds different from those used to diagnose ID should be used to monitor the need for re-dosing. Creating efficient and effective care pathways that can be audited is a major challenge. Measuring serum ferritin causes more confusion than clarity, although new ferritin assays might change this perspective [48].

Some markers and thresholds are widely used, particularly TSAT < 20% in the diagnosis of ID, and correspondingly TSAT ≥ 20% as an important treatment target. Most agreed that ID was uncommon in the absence of anaemia or borderline anaemia (0–10 g/L above current WHO criteria). The utility of serum iron and ferritin was controversial.

As such, the panel agreed that additional research in this area was needed. There are a few placebo-controlled trials of iron therapy in CKD. Several panellists made explicit calls for further trials in a range of different areas:

- “There’s lots of extra renal and extra cardiac effects that we should be thinking about…there’s a lot of research to go on yet.”
- “We don’t have a primary outcome study showing quality of life measures, only secondary outcomes. There’s some suggestion that may be beneficial, but it’s not proven as yet.”
- “A trial needs to be done to test a lower [treatment threshold for] ferritin of 700 and higher of 1400. I’d love to randomise low clearance patients to iron or no iron way before they have ESAs.”
- “What’s clearly lacking in the renal space is a decent, long-term, non-dialysis CKD study.”

Clearly, there is a need to consolidate the various current approaches to diagnosis and treatment of ID in people with CKD and/or HF. Simple, clear strategies and messages are key. Simple markers of ID and thresholds for managing iron deficiency are required.

## Data Availability

Raw data from the Stage 1 questionnaire and Stage 3 poll results will be made available upon request to the corresponding author: Harry Lewis, h.lewis@initiateconsultancy.com.

## Supplementary Materials

The following supporting information can be downloaded at: https://www.mdpi.com/article/10.3390/jcm15041676/s1, File S1: Recreation of questionnaire.

## Abbreviations

The following abbreviations are used in this manuscript:

CHr	Reticulocyte haemoglobin content
CKD	Chronic kidney disease
ESA	Erythropoietin stimulating agent
ESC	European Society of Cardiology
GI	Gastrointestinal
HF	Heart failure
HRC	Hypochromic red cells
ID	Iron deficiency
IDA	Iron deficiency anaemia
IV	Intravenous
KDIGO	Kidney Disease Improving Global Outcomes
NICE	National Institute of Health and Care Excellence
sTfR	Soluble transferrin receptor
TSAT	Transferrin saturation
WHO	World Health Organisation
