# Supplementary material for: Expert Perspectives on Managing Iron Deficiency in People with CKD and/or HF
Source: J Clin Med. 2026 Feb 23;15(4):1676. doi: 10.3390/jcm15041676 (PMC12941927; doi:10.3390/jcm15041676)
Supplement: Supplementary file 1 [file jcm-15-01676-s001.zip › jcm-4076733-supplementary.pdf]

Recreation of questionnaire

Q1. To start this survey, please enter your name and occupation.

____

Q2. How many years experience do you have managing iron deficiency?

____

Q3. Please estimate how many people with iron deficiency are in your centre.

____

Q4. Please estimate how many people with iron deficiency you personally manage.

____

Q5. Please estimate how many new people with iron deficiency you would see annually.

____

Q6. Who is currently involved in the diagnosis, treatment, and management of people with iron deficiency at your centre?

	Involved (tick all that apply)	Number of team members (estimated)	Responsibilities
Primary care (pharmacist)	◻		
Primary care (GP)	◻		
Primary care (nurse)	◻		
Secondary care (consultant nephrologist/cardiologist)	◻		
Secondary care (nurse)	◻		
Secondary care (nutritionist)	◻		
Psychological care (psychologist)	◻		
Psychological care (counselling)	◻		
Self-care (self-management courses)	◻		
Self-care (other support)	◻		
Other (please list)	◻		

Q7. Should people be screened for iron deficiency independent of anaemia?

◻ Yes

◻ No

Q8. If you answered yes to the previous question, what is the testing frequency for iron deficiency that is recommended, e.g., per month? If you answered no, what is the threshold Hb of untreated anaemia that would trigger an investigation into iron deficiency?

____

Q9. Please select how often you would refer to each guideline when diagnosing iron deficiency.

	How often the guideline is referred to			
	Never	Occasionally	Often	Always
‘Kidney disease: Improving global outcomes (KDIGO) anemia work group. KDIGO clinical practice guideline for anemia in chronic kidney disease’, 2012, https://kdigo.org/wp-content/uploads/2016/10/KDIGO-2012-Anemia-Guideline-English.pdf	☐	☐	☐	☐
‘Controversies in optimal anemia management: conclusions from a Kidney Disease: Improving Global Outcomes (KDIGO) Conference’, 2021, https://www.kidney-international.org/article/S0085-2538(21)00355-0/fulltext	☐	☐	☐	☐
‘2021 ESC Guidelines for the diagnosis and treatment of acute and chronic heart failure’, 2021, https://www.escardio.org/Guidelines/Clinical-Practice-Guidelines/Acute-and-Chronic-Heart-Failure	☐	☐	☐	☐
‘2023 Focus Update of the 2021 ESC Guidelines for the diagnosis and treatment of acute and chronic heart failure’, 2023, https://www.escardio.org/Guidelines/Clinical-Practice-Guidelines/Focused-Update-on-Heart-Failure-Guidelines	☐	☐	☐	☐
‘Chronic kidney disease: assessment and management’ by NICE, 2021, https://www.nice.org.uk/guidance/ng203/resources/chronic-kidney-disease-assessment-and-management-pdf-66143713055173	☐	☐	☐	☐
Clinical Practice Guideline Anemia of Chronic Kidney Disease, 2022, https://ukkidney.org/sites/renal.org/files/Updated130220-Anaemia-of-Chronic-Kidney-Disease-1-1.pdf	☐	☐	☐	☐
‘Kidney Disease: Improving Global Outcomes guidelines on anaemia management in chronic kidney disease: a European Renal Best Practice position statement’, 2013, https://academic.oup.com/ndt/article/28/6/1346/1839326?llogin=false	☐	☐	☐	☐

Other (please list any other guidelines you use)	☐	☐	☐	☐

Q10. What parameters would you use to evaluate iron deficiency, and in what order of importance?

	Threshold/ parameter	Importance of marker for indicating iron deficiency			
	Please add	Important/always use	Important/sometimes use	Not useful	Useful but not available in my practice
Serum ferritin (μg/dL)		☐	☐	☐	☐
Transferrin saturation (%)		☐	☐	☐	☐
Transferrin (mg/L)		☐	☐	☐	☐
HRC (hypochromic red cells) (%)		☐	☐	☐	☐
CHr (reticulocyte Hb content) (pg/cell)		☐	☐	☐	☐
Soluble transferrin receptor (sTFR) (mg/L)		☐	☐	☐	☐
Serum iron (μg/dL)		☐	☐	☐	☐
Haemoglobin (g/L)		☐	☐	☐	☐
Haematocrit (%)		☐	☐	☐	☐
Other (please list)		☐	☐	☐	☐

Q11. Please select how often you would refer to each guideline when <u>treating</u> iron deficiency.

	How often the guideline is referred to			
	Never	Occasionally	Often	Always
'Kidney disease: Improving global outcomes (KDIGO) anemia work group. KDIGO clinical practice guideline for anemia in chronic kidney disease', 2012, https://kdigo.org/wp-content/uploads/2016/10/KDIGO-2012-Anemia-Guideline-English.pdf	☐	☐	☐	☐
'Controversies in optimal anemia management: conclusions from a Kidney Disease: Improving Global Outcomes (KDIGO) Conference', 2021, https://www.kidney-international.org/article/S0085-2538(21)00355-0/fulltext	☐	☐	☐	☐
'2021 ESC Guidelines for the diagnosis and treatment of acute and chronic heart failure', 2021,	☐	☐	☐	☐

https://www.escardio.org/Guidelines/Clinical-Practice-Guidelines/Acute-and-Chronic-Heart-Failure				
‘2023 Focus Update of the 2021 ESC Guidelines for the diagnosis and treatment of acute and chronic heart failure’, 2023, https://www.escardio.org/Guidelines/Clinical-Practice-Guidelines/Focused-Update-on-Heart-Failure-Guidelines	☐	☐	☐	☐
‘Chronic kidney disease: assessment and management’ by NICE, 2021, https://www.nice.org.uk/guidance/ng203/resources/chronic-kidney-disease-assessment-and-management-pdf-66143713055173	☐	☐	☐	☐
Clinical Practice Guideline Anemia of Chronic Kidney Disease, 2022, https://ukkidney.org/sites/renal.org/files/Updated130220-Anaemia-of-Chronic-Kidney-Disease-1-1.pdf	☐	☐	☐	☐
‘Kidney Disease: Improving Global Outcomes guidelines on anaemia management in chronic kidney disease: a European Renal Best Practice position statement’, 2013, https://academic.oup.com/ndt/article/28/6/1346/1839326?llogin=false	☐	☐	☐	☐
Other (please list any other guidelines you use)	☐	☐	☐	☐

Q12. What proportion (%) of people have their iron deficiency proactively managed, i.e., you regularly monitor for iron deficiency irrespective of anaemia and treat iron deficiency when diagnosed?

____

Q13. What proportion (%) of people have their iron deficiency reactively managed, i.e., you only screen for iron deficiency when patients complain of symptoms or if they have anaemia/low haemoglobin?

____

Q14. What is the expected timeline for seeing improvement in iron levels/symptoms after starting treatment?

____

Q15. Please rate (scale of 1-5, 1 being unsuccessful and 5 being successful) how successful current treatment approaches are for:

	1	2	3	4	5

Managing iron deficiency	☐	☐	☐	☐	☐
Achieving optimal iron level thresholds	☐	☐	☐	☐	☐
Maintaining targets	☐	☐	☐	☐	☐
Improving patient outcomes and symptoms	☐	☐	☐	☐	☐

Q16. Please list the optimal target you would like your patient to achieve with treatment of iron deficiency:

	Optimal target/level to achieve after treatment
	Please add
Serum ferritin (µg/dL)	
Transferrin saturation (%)	
Transferrin (mg/L)	
HRC (hypochromic red cells) (%)	
CHr (reticulocyte Hb content) (pg/cell)	
Soluble transferrin receptor (sTFR) (mg/L)	
Serum iron (µg/dL)	
Haemoglobin (g/L)	
Haematocrit (%)	
Other (please list any other markers you would investigate)	

Q17. Please state the recommended frequency of monitoring iron status tests.

____

Q18. Are there upper limits of iron status tests beyond which iron therapy shows little or no benefit, evidence of risk, or both?

	Upper limit (if applicable)
	Please add or write in NA
Serum ferritin (µg/dL)	
Transferrin saturation (%)	
Transferrin (mg/L)	
HRC (hypochromic red cells) (%)	
CHr (reticulocyte Hb content) (pg/cell)	
Soluble transferrin receptor (sTFR) (mg/L)	
Serum iron (µg/dL)	
Haemoglobin (g/L)	
Haematocrit (%)	
Other (please list any other markers you would investigate)	

Q19. What proportion (%) of people at your centre have uncontrolled iron deficiency despite compliant use of standard therapy?

____

Q20. Statement pieces (please tick how strongly you agree/disagree with each statement)

	Strongly disagree	Somewhat disagree	Neither agree nor disagree	Somewhat agree	Strongly agree
1. People with chronic kidney disease or heart failure are at increased risk of iron deficiency.	☐	☐	☐	☐	☐

2. Iron deficiency in people with heart failure or chronic kidney disease is underdiagnosed.	☐	☐	☐	☐	☐
3. Iron deficiency in people with heart failure or chronic kidney disease is undertreated.	☐	☐	☐	☐	☐
4. The common symptoms of iron deficiency can vary significantly across different age groups and genders.	☐	☐	☐	☐	☐
5. Treating iron deficiency irrespective of anaemia is important to prevent complications and improve patient outcomes.	☐	☐	☐	☐	☐
6. Early diagnosis and management of iron deficiency are crucial to prevent complications and improve patient outcomes.	☐	☐	☐	☐	☐
7. Consideration of markers beyond standard blood tests may be necessary in certain situations to evaluate iron status accordingly.	☐	☐	☐	☐	☐
8. Chronic diseases such as heart failure or chronic kidney disease can complicate the interpretation of iron markers.	☐	☐	☐	☐	☐
9. Iron deficiency can be effectively managed with existing treatments.	☐	☐	☐	☐	☐
10. Treatment targets and diagnosis thresholds currently vary for specific patient populations, such as individuals with chronic kidney disease or heart failure.	☐	☐	☐	☐	☐
11. Treatment targets and diagnosis thresholds should differ between different patient populations, such as chronic kidney disease or heart failure.	☐	☐	☐	☐	☐
12. Individuals with chronic kidney disease or heart failure should receive specialised management for iron deficiency.	☐	☐	☐	☐	☐
13. There is a need for consistent thresholds for defining and targets for treating iron deficiency among sub-specialties.	☐	☐	☐	☐	☐

14. Continuous research and clinical trials are needed to further refine and improve the management of iron deficiency and related conditions.	☐	☐	☐	☐	☐
